# Remote Estimation of Rice Yield With Unmanned Aerial Vehicle (UAV) Data and Spectral Mixture Analysis

**DOI:** 10.3389/fpls.2019.00204

**Published:** 2019-02-27

**Authors:** Bo Duan, Shenghui Fang, Renshan Zhu, Xianting Wu, Shanqin Wang, Yan Gong, Yi Peng

**Affiliations:** ^1^School of Remote Sensing and Information Engineering, Wuhan University, Wuhan, China; ^2^Lab for Remote Sensing of Crop Phenotyping, Wuhan University, Wuhan, China; ^3^College of Life Sciences, Wuhan University, Wuhan, China; ^4^College of Resource and Environment, Huazhong Agricultural University, Wuhan, China

**Keywords:** rice, yield, remote sensing (RS), unmanned aerial vehicle (UAV), vegetation index (VI), spectral mixture analysis (SMA)

## Abstract

The accurate assessment of rice yield is crucially important for China’s food security and sustainable development. Remote sensing (RS), as an emerging technology, is expected to be useful for rice yield estimation especially at regional scales. With the development of unmanned aerial vehicles (UAVs), a novel approach for RS has been provided, and it is possible to acquire high spatio-temporal resolution imagery on a regional scale. Previous reports have shown that the predictive ability of vegetation index (VI) decreased under the influence of panicle emergence during the later stages of rice growth. In this study, a new approach which integrated UAV-based VI and abundance information obtained from spectral mixture analysis (SMA) was established to improve the estimation accuracy of rice yield at heading stage. The six-band image of all studied rice plots was collected by a camera system mounted on an UAV at booting stage and heading stage respectively. And the corresponding ground measured data was also acquired at the same time. The relationship of several widely-used VIs and Rice Yield was tested at these two stages and a relatively weaker correlation between VI and yield was found at heading stage. In order to improve the estimation accuracy of rice yield at heading stage, the plot-level abundance of panicle, leaf and soil, indicating the fraction of different components within the plot, was derived from SMA on the six-band image and *in situ* endmember spectra collected for different components. The results showed that VI incorporated with abundance information exhibited a better predictive ability for yield than VI alone. And the product of VI and the difference of leaf abundance and panicle abundance was the most accurate index to reliably estimate yield for rice under different nitrogen treatments at heading stage with the coefficient of determination reaching 0.6 and estimation error below 10%.

## Introduction

Rice (*Oryza sativa* L.) is one of the most important grain crops in the world, especially in China. There are more than half of the China’s population regarding rice as the staple food (Xiao et al., 2002). The accurate estimation of rice yield is of significance to ensure food security and promote sustainable development.

Remote sensing (RS) is a technique which can obtain the information about an object without making physical contact with the object (Wikipedia^1^) and it can efficiently obtain canopy spectra data in a non-destructive way, which carries valuable information indicating the canopy interaction with solar radiation such as vegetation absorption and scattering (Thenkabail et al., 2011). The vegetation canopy spectra are closely related to vegetation growth. In the visible range, vegetation has strongly absorption of light due to the pigments and presents as low reflectance (Woolley, 1971). But in the near-infrared (NIR) range, vegetation reflectance is relatively high and affected by thick plant tissues and canopy structure (Gausman et al., 1969). A series of studies have been developed to relate the vegetation spectra to vegetation growth parameters such as chlorophyll content (Gitelson et al., 2006; Wu et al., 2008; Feret et al., 2011), leaf area index (LAI) (Broge and Leblanc, 2001; Viña et al., 2011) and biomass (Thenkabail et al., 2000; Hansen and Schjoerring, 2003), and thus a lot of vegetation indices (VIs) calculated from reflectance of different spectra ranges (Hatfield et al., 2008) have been proposed to accurately estimate these parameters. For example, Peng et al. (2013) used Enhanced Vegetation Index (EVI) and Wide Dynamic Range Vegetation Index (WDRVI) obtained from MODIS to accurately estimate the gross primary productivity in crops with coefficients of variation below 20% in maize and 25% in soybean (Peng et al., 2013). Li et al. (2014) applied several red-edge based spectral indices to estimate plant nitrogen uptake with the coefficient of determination above 0.76. Parametric statistical approaches based on VIs are by far the simplest and most studied variable estimation approaches and have been widely used in monitoring crop growth (Verrelst et al., 2015). And the changes of crop growth status, which can be effectively monitored by spectral measures, directly determine its ultimate yield. Therefore, VIs have also exhibited good potential in remote estimation of crop yield especially at the large scales (Rahman et al., 2012; Sun et al., 2017). Becker-Reshef et al. (2010) estimated the wheat yield in Kansas and Ukraine with a 7% error by using time series Normalized Difference Vegetation Index (NDVI) data from MODIS. Holzman et al. (2014) came up with a new method to estimate regional crop yield using the Temperature Vegetation Dryness Index (TVDI) with the RMSE values ranged from 12 to 13% for soybean and 14 to 22% for wheat. Sakamoto et al. (2014) mapped U.S. corn yields successfully using Wide Dynamic Range Vegetation Index (WDRVI) derived from timeseries MODIS data with the estimation error below 30% at the state level. Generally, VI-based methods are the mainstream approach for crop yield prediction, and a lot of regression algorithms of using VIs to estimate crop yield were established including simple linear functions and complex non-linear functions (Tucker et al., 1980). Many experiments showed that the use of appropriate VI was the key to crop yield estimation instead of the complex function structures in VI-based methods (Gholizadeh et al., 2015). Therefore, it is particularly important for crop yield evaluation to get the exact VIs.

However, there may be a considerable discrepancy between pixel sizes of RS images (e.g., 1 km for MODIS satellite image) and much smaller sizes of croplands (e.g., usually 33 m × 20 m in South China) due to the limitation of the spatial resolution as well as the landscape fragmentation (Gong et al., 2018). In this case, one pixel of a RS image may contain several land cover types and the signal of this pixel (mixed pixel) is the outcome of various land cover components that have significantly different spectra. VI, derived from the spectra of such mixed pixels, may encompass the unexpected information of the components not related to yield, which could lower the precision of yield estimation. The mixed pixel is always a problem that have to be discussed in the application of RS technique. Even for the high spatial resolution images, the problem of mixed pixel still exists because of the smaller cropland components such as flower, fruit and grain. This problem is more obvious when addressing rice yield prediction with RS data in VI-based method. Rice is a type of grain crop, the panicle will gradually emerge in paddy rice field when rice steps into reproductive phase and may last until maturation. In the early stage of rice panicle emergence, the panicle was bright green and scarcely distributed in rice canopy, while rice leaves were still the dominant component in paddy rice field. With the continuous growth of rice, the amount of panicle increased and its color gradually changed to yellow. Since rice panicle had the dramatically different spectra from rice leaves, the remotely detected canopy spectra of rice after panicle emergence were greatly mixed by panicle and leaf spectra, and the accuracy of estimated crop growth parameters in VI-based method would decrease. Zhou et al. (2017) found that the appearance of the rice panicle enhances the difficulty of LAI estimation and yield prediction in the later growth stages of rice. And Sakamoto et al. (2011) found the same result in rice that the color index performed well-before the booting stage but poorly in the late growth stages. Harrell et al. (2011) reported that the reduced predictive ability at heading stage was most likely associated with the uneven emergence of panicles into the sensor field of view. These studies showed that the canopy spectra remotely obtained from rice in late growth stages were susceptible to the interference of uneven emergence of panicles, and the factor of spectral mixture that influence the rice yield estimation must be considered.

Spectral mixture analysis (SMA) was extensively used to quantify the spectral contributions from different components in a mixed pixel. It assumed that the spectrum of a mixed pixel was a linear or non-linear combination of its constituent spectral components (called endmember) weighted by their subpixel fractional cover (called abundance) (Somers et al., 2011). Once the pure spectra of endmembers were obtained, the fraction of each endmember within a mixed pixel can be estimated based on its mixed spectra (Bioucas-Dias et al., 2012). SMA has been widely applied in RS for evaluating vegetation properties. Gitelson et al. (2002) developed an approach to estimate vegetation fraction in sampling zones based on measured spectra of two endmembers (bare soil and dense vegetation). Lobell and Asner presented a SMA approach that employs time series of MODIS data to estimate subpixel fractions of land cover types with R^2^ reaching 0.8 (Lobell and Asner, 2004). Therefore, SMA can be a good tool to analyze the influence of spectral mixture to rice yield estimation and it also has good potential to improve the accuracy of rice yield estimation by combining with VIs.

In recent years, unmanned aerial vehicles (UAVs) have become an increasingly used platform for RS application in Precision agriculture due to its high spatial and temporal resolutions (Zarcotejada et al., 2013; Candiago et al., 2015; Fang et al., 2016; Aasen and Bolten, 2018). And the UAV-collected data is playing a more and more important role in monitoring crop growth. Based on multi-band images obtained by an UAV system, this study explores to improve VI-based approach for rice yield estimation by combining with SMA.

## Materials and Methods

### Study Area

**FIGURE 1:**
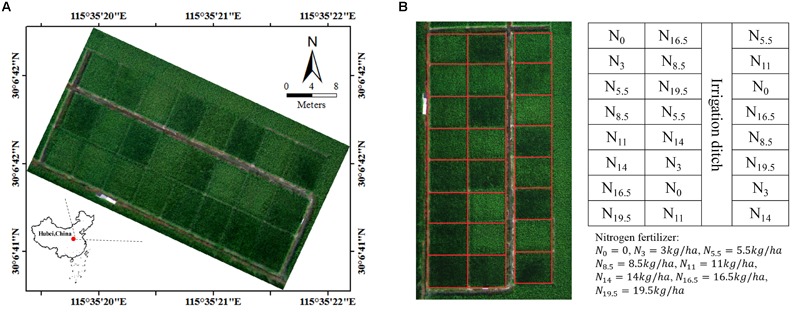
**(A)** the location of study site and **(B)** the level of nitrogen fertilizer in each plot.

The study site was located at the Rice Experiment and Research Base of Huazhong Agricultural University near Wuxue City, Hubei Province, China (30.1117°N, 115.5892°E). In this investigation, the data from 24 rice plots was studied – Figure 1A. They were of the size about 20 m^2^ including around 330 plants and all planted with the same hybrid of rice. The field management for these plots were similar except that different levels of nitrogen fertilizer were applied. Eight levels of nitrogen fertilizer (0, 3, 5.5, 8.5, 11, 14, 16.5, and 19.5 kg/ha) were utilized, and each level was repeated on three randomly distributed plots – Figure 1B. The growing period for rice in our study was from June to September, and field experiments were conducted during booting (13 August, 2015) and heading (29 August, 2015) stages of rice growth. At these two stages, rice has gradually completed the transformation from vegetative period to reproductive period. There was no obvious panicle in rice field at booting stage; on the contrary, the panicle began to emerge at heading stage. During the course of each experiment, one UAV flight was arranged to obtain the image of all rice plots. After the UAV flight (from 11:00 am to 2:00 pm), the corresponding ground measurements were carried out *in situ* immediately.

### Field Data Collection

The LAI and leaf chlorophyll content were measured at booting (13 August, 2015) and heading (29 August, 2015) stage of rice growth. Ground rice canopy LAI was measured using a SunScan canopy analysis system (Delta-T Devices, Ltd., Burwell, Cambridge, United Kingdom) under windless conditions and stable light levels. For each plot, three LAI readings were acquired and their average represented the canopy LAI of the plot. A SPAD-502 Chlorophyll Meter (Soil and Plant Analyzer Development Chlorophyll Meter, Spectrum Technologies, Inc., Plainfield, IL, United States; abbreviated as SPAD) was utilized to measure the leaf chlorophyll content of rice. Five positions were selected in each plot, and the rice leaf chlorophyll content was measured in each position. For each position in plot, three SPAD values of top layer leaf, middle layer leaf, and bottom layer leaf were recorded and the average SPAD indicated the rice leaf chlorophyll content of the position. And the plot level leaf chlorophyll content was the average of five SAPD values in five positions.

At the heading stage of rice, some samples of typical ground features were collected and their spectra were measured as the endmember spectra used for SMA. The spectra of six kinds of endmember were measured using an ASD Field Spec 4 spectrometer (Analytical Spectral Devices Inc., Boulder, CO, United States), including top layer leaf (TL), bottom layer leaf (BL), top layer panicle (TP), bottom layer panicle (BP), dry soil (DS), and wet soil (WS). Samples of different endmember were collected from the studied area and their spectra were measured *in situ* immediately, and the measurements were carried out in a stable light condition after UAV flight. For soil spectra collection, we walked into the rice field and selected several representative sample-collecting spots in the ridges between plots. The measurement of soil spectra was conducted using ASD probe pointing to the ground vertically at the appropriate height to make sure the field of view was full of wet or dry soil with no other land cover features, and the averaged spectra were used as soil spectra. In the same way, the panicle spectra were obtained with ASD probe pointing downward approximately 10 cm above the panicle samples. Since the rice panicle was small and granular, the samples of panicle were put on a black background and evenly spread. As for the leaf spectra, a self-illuminated leaf clip of ASD was used and then the spectra of top layer leaf and bottom layer leaf were taken respectively. In the whole study area, five positions were selected randomly and the spectra of top layer leaf, bottom layer leaf, top layer panicle, and bottom layer panicle were gained respectively for each plot. Similarly, the averaged spectra were used as their endmember spectra.

At maturity, the all rice plants in each plot were harvested manually for determination of grain yield. The seeds were cleaned and exposed to the sun until their weight did not change. And then all the dry seeds were weighted according to plots and the plot-level yield was obtained.

### Surface Reflectance and Vegetation Index Derived From UAV Data

**FIGURE 2:**
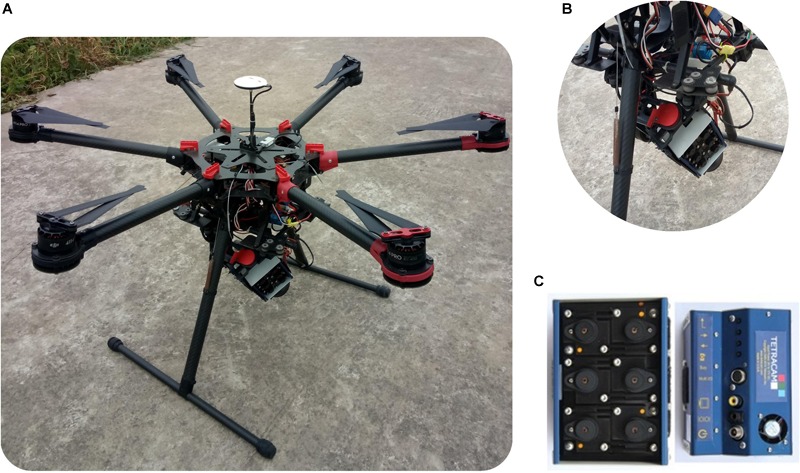
The illustration of **(A)** UAV, **(B)** gimbal, and **(C)** Mini-MCA.

The image of study plots was acquired using a Mini-MCA system mounted on a UAV (S1000, SZ DJI Technology, Co., Ltd., Shenzhen, China) on 13 August and 29 August, 2015 – Figure 2. The Mini-MCA system consists of an array of six individual miniature digital cameras (Mini-MCA 6, Tetracam, Inc., Chatsworth, CA, United States) – Figure 2C. Each camera imager was equipped with a customer-specified band pass filter centered at a wavelength of 490, 550, 670, 720, 800, or 900 nm, respectively, and the band width was 10 nm. These bands were selected since they were commonly employed to analyze vegetation growth-related parameters (Behrens et al., 2006; Ray et al., 2010; Kira et al., 2015).

The Mini-MCA system was attached to the UAV on a gimbal which can help to compensate for the UAV movement (pitch and roll) during the flight and guarantee close to nadir image collection – Figure 2B (Turner et al., 2014). Since the imaging system had a significant camera mis-registration effect, six cameras were co-registered in the laboratory prior to the flight so that corresponding pixels of each lens were spatially overlapping in the same focal plane (Jhan et al., 2016). UAV flight was conducted under clear skies with little cloud cover between 10:00 and 14:00 local time when the changes in the solar zenith angle were minimal. The image was collected at approximately 60 m above the ground with the spatial resolution of 3 cm around.

In this study, an empirical linear correction method was applied to transform image Digital Numbers (DNs) into surface Reflectance (ρ) (Dwyer et al., 1995; Laliberte et al., 2011). Four calibration ground targets were placed in the cameras’ field of view as a standard for image radiometric corrections and the image taken from Mini-MCA system included all of them. The calibration targets used in this study have the relatively constant reflectance of 0.06, 0.24, 0.48, and 1 respectively throughout the visible to NIR wavelengths and are specially utilized for aerial image radiometric calibration. As a linear relationship was assumed between DNs and ρ, the canopy surface reflectance can be calculated as

(1) $$\rho_{\lambda }=DN_{\lambda }\times Gain_{\lambda }+Offset_{\lambda }(\lambda =490,550,\;670,\;720,\;800\;and\;900\;nm)$$

Where ρ_λ_ and DN_λ_ are the surface reflectance and digital number, respectively, of a given pixel at wavelength λ; Gain_λ_; and Offset_λ_ are gains and bias of the camera at different wavelengths respectively. For each wavelength, Gain_λ_ and Offset_λ_ can be calculated using the least-square method from ρ and DN values (referring to DN_0.06_, DN_0.24_, DN_0.48_, and DN_1_) of four calibration targets.

(2) $$\left(\begin{array}{c} 0.06\\ 0.24\\ 0.48\\ 1 \end{array}\right)=\left(\begin{array}{c} DN_{0.06}\\ DN_{0.24}\\ DN_{0.48}\\ DN_{1} \end{array}\right)\times Gain_{\lambda }+Offset_{\lambda }$$

(3) $$\left(\begin{array}{c} Offset_{\lambda }\\ Gain_{\lambda } \end{array}\right)={\left[{\left(\begin{array}{cc} 1 & DN_{0.06}\\ 1 & DN_{0.24}\\ 1 & DN_{0.48}\\ 1 & DN_{1} \end{array}\right)}^{T}\left(\begin{array}{cc} 1 & DN_{0.06}\\ 1 & DN_{0.24}\\ 1 & DN_{0.48}\\ 1 & DN_{1} \end{array}\right)\right]}^{-1}{\left(\begin{array}{cc} 1 & DN_{0.06}\\ 1 & DN_{0.24}\\ 1 & DN_{0.48}\\ 1 & DN_{1} \end{array}\right)}^{T}\left(\begin{array}{c} 0.06\\ 0.24\\ 0.48\\ 1 \end{array}\right)$$

For each of 24 plots, we defined a maximum rectangle in the image that fit the plot – Figure 1B. The rectangle included approximated 18000 pixels, and the plot-level VI was retrieved by averaging all of the per-pixel values within the rectangle. The VI tested in this study are shown in Table 1.

**Table 1:** Vegetation Indices tested in this study.

Vegetation Indices	Formula	Reference
Simple Ratio (SR)	ρ_800_/ρ_670_	Jordan, 1969
Red-edge Chlorophyll Index (CI_rededge_)	ρ_800_/ρ_720_ - 1	Gitelson et al., 2005
Green-edge Chlorophyll Index (CI_green_)	ρ_800_/ρ_550_ - 1	Gitelson et al., 2005
Normalized Difference Vegetation Index (NDVI)	(ρ_800_ - ρ_670_)/(ρ_800_ + ρ_670_)	Rouse et al., 1974
Green Normalized Difference Vegetation Index (GNDVI)	(ρ_800_ - ρ_550_)/(ρ_800_ + ρ_550_)	Gitelson et al., 1996
Normalized Difference Red edge (NDRE)	(ρ_800_ - ρ_720_)/(ρ_800_ + ρ_720_)	Glenn et al., 2010)
Visible Atmospherically Resistant Index (VARI)	(ρ_550_ - ρ_670_)/(ρ_550_ + ρ_670_)	Gitelson et al., 2002
MERIS Terrestrial Chlorophyll Index (MTCI)	(ρ_800_ - ρ_720_)/(ρ_720_ - ρ_670_)	Dash and Curran, 2004
Enhanced Vegetation Index (EVI)	2.5(ρ_800_ - ρ_670_)/(ρ_800_ + 6ρ_670_ - 7.5ρ_490_ + 1)	Liu and Huete, 1995
Two-band Enhanced Vegetation Index (EVI2)	2.5(ρ_800_ - ρ_670_)/(ρ_800_ + 2.4ρ_670_ + 1)	Jiang et al., 2008

### Fully Constrained Least Squares Linear Spectral Mixture Analysis

**FIGURE 3:**
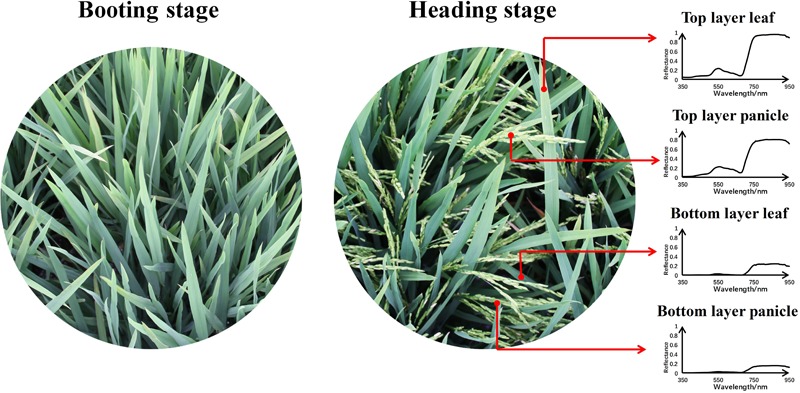
The actual scene of paddy field at booting stage and heading stage.

**FIGURE 4:**
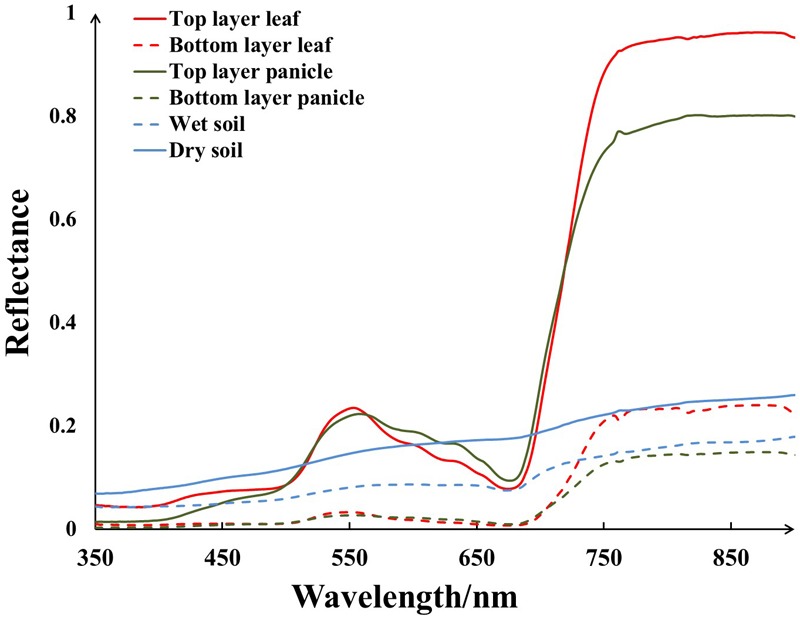
The ground measured spectra of selected endmembers.

Although UAV made it possible to get high resolution data, one pixel on a UAV image still encompassed several land cover components especially for crops with distinct grains like rice. When rice was at heading stage, there were six dominant components visible in the field of view – Figure 3, including top layer leaf (TL), bottom layer leaf (BL), top layer panicle (TP), bottom layer panicle (BP), dry soil (DS), and wet soil (WS). And their continuous spectra were collected by an ASD spectrometer used as the endmember spectra for SMA – Figure 4. Compared with the ground measured reflectance spectra, the spectra acquired by the MCA camera onboard UAV were discrete wavebands (490, 550, 670, 720, 800, and 900 nm, 10 nm band width). For each endmember, with reference to the wavelength range of each MCA waveband, the average of ground measured endmember reflectance in the corresponding wavelength range was calculated as the endmember reflectance used for the SMA of UAV images. Therefore, six kinds of endmember reflectance were obtained as: ρ(TL), ρ(BL), ρ(TP), ρ(BP), ρ(DS), and ρ(WS).

In this study, fully constrained least squares linear SMA method was employed to estimate the abundance fractions of components present in an image pixel. For linear spectral mixing model, a mixed pixel was defined as a linear combination of components with their relative concentrations (Heinz, 2001). In this case, the reflectance ρ of a mixed pixel at wavelength λ can be approximated as

(4) $$\rho_{\lambda }=\sum_{i=1}^{N}Abd_{i}\;\rho_{\lambda }(i)+e$$

where e is noise or can be interpreted as a measurement error, N is the number of selected endmembers, Abd_i_ denotes the abundance fraction of endmember i, and ρ_λ_(i) is the reference reflectance of endmember i at wavelength λ.

And for the fully constrained linear mixing model, two partially constraints were considered which were referred to as non-negatively constraint and sum-to-one constraint

(5) $$0\leq Abd_{i}\leq 1;\text{and}\sum_{i=1}^{N}Abd_{i}\text{=1}$$

As shown above, the value of abundance was non-negative with its maximum constrained to 1 and for each mixed pixel the sum of the endmember abundance equals to 1. Generally, the abundance that meets these conditions can indicate the proportion of the corresponding endmember within the mixed pixel. An abundance of 0 means no presence of the particular endmember in this pixel, while an abundance of 1 indicates that this pixel is a pure pixel of the particular endmember.

To solve the abundance of these selected six components, a least squares method was applied (Pu et al., 2014). In this study, we used MATLAB (MATLAB 2016a, MathWorks, Inc., Natick, MA, United States) to derive the abundance gray images of six selected endmembers whose gray level values meant the estimated abundance of the corresponding endmember in the image pixel. The plot-level abundance was calculated in the same manner as plot-level reflectance, and for each plot the same maximum rectangle was utilized again. In consideration of endmember selection, the panicle abundance (Abd_P_) and leaf abundance (Abd_L_) were the sum of the corresponding elements in top layer and bottom layer together.

### Data Analysis Among UAV Data, Ground Measured Data, and Rice Yield

In this study, the IBM SPSS Statistics (Statistical Product and Service Solutions 22.0, IBM, Armonk, NY, United States) was used to statistically describe and analyze data. Firstly, a normally distribution test was applied to the rice yield data and the product data of LAI and SPAD value (LAI × SPAD) at booting and heading stage. The result of Shapiro–Wilk test served as the indicator to test whether the data is normally distributed or not. And then, the correlation analysis and regression analysis were successively applied. The Pearson correlation coefficient (r) was exhibited as the result of correlation analysis. And for regression analysis, adjusted R square (R^2^), root mean square error (RMSE) and *p*-value were analyzed and compared. The detailed calculation method of above evaluation indices can be found in the help document of SPSS.

The relationship between ground measured data (LAI × SPAD) and rice yield was firstly established at booting and heading stage respectively. And the plot-level VI derived from UAV data was correlated with rice yield directly and compared with the ground measured data. The difference of these two rice growth stages was discussed and analyzed.

At the heading stage of rice, in consideration of the impact of spectral mixture within 1 pixel, the leaf and panicle make different spectral contributions to the reflectance of the mixed pixel. For each studied plot, different proportion of leaf and panicle may affect the accuracy of yield estimation based on plot-level VI at heading stage. In order to eliminate the influence, plot-level VI was incorporated with abundance information to relate with rice yield and four relationships were developed using 23 samples (one of the rice yield data was obviously wrong): (1) yield vs. VI, (2) yield vs. VI × Abd_L_, (3) yield vs. VI × Abd_P_ and (4) yield vs. VI × Abd_L-P_. The different estimation ability for rice yield of these four kinds of index was compared and evaluated.

### Algorithm Establishment Using Leave One Out Cross-Validation

The final rice yield estimation model was established using a leave one out cross-validation method. Leave one out cross-validation is s statistics method widely applied in model establishment and validation (Fielding and Bell, 1997). It divided the samples into two groups, one for training and the other one is used for validation. For leave one out cross-validation, the validation set just included one sample and the training and validating process was repeated K times (K equals to the number of samples, *K* = 23 in this study). For each time i, K-1 samples were used iteratively as training data to calibrate the coefficients (Coef_i_) of the algorithm with the accuracy was measured in terms of coefficients of determination ($R_{i}^{2}$), and the remaining single sample was used for validation to obtain the estimation error (E_i_). The training and validating process was repeated K times until every single sample was used exactly one time for validation data. After K iterations, the coefficients and accuracy of the final algorithm can be produced as

$$Coef=\frac{\sum_{i=1}^{k}Coef_{i}}{K}\;R^{2}=\frac{\sum_{i=1}^{k}R_{i}^{2}}{K}\;RMSE=\sqrt{\frac{\sum_{i=1}^{k}E_{i}^{2}}{K}}$$

## Results

### Relationship Between Ground Measured Data and Rice Yield

**FIGURE 5:**
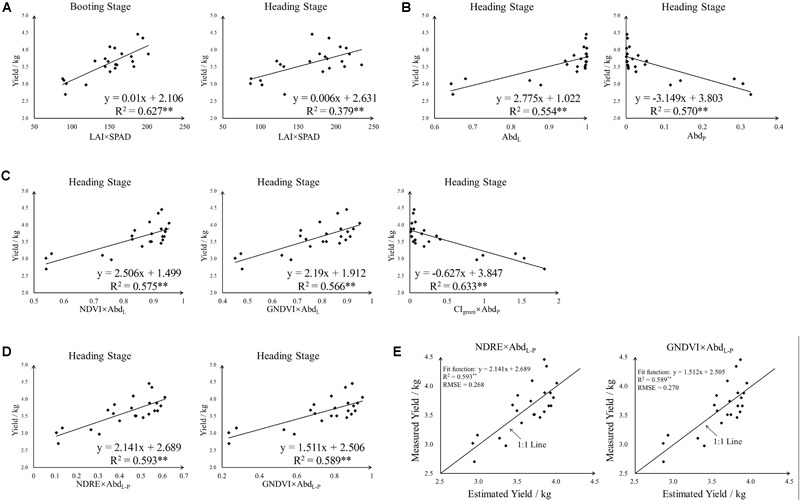
The linear regression result of yield and different indices. **(A)** Yield vs. LAI × SPAD, **(B)** yield vs. abundance, **(C,D)** yield vs. the product of VI and abundance and **(E)** Estimated yield vs. measured yield. ^∗∗^*F*-test statistical significance at 0.01 probability level.

In general, the product of LAI and leaf-level SPAD (LAI × SPAD) was used to estimate canopy chlorophyll status of rice and has been proven to be a promising index to predict rice yield (Liu et al., 2017). In this study, LAI × SPAD was calculated according to the ground measured LAI and SPAD value both at booting and heading stage. Among all plots which have yield data, one of the plot-level LAI records was missing and 22 LAI × SPAD values was calculated at these two stages. Before correlation analysis and regression analysis, a normal distribution test was applied to the rice yield data and LAI × SPAD data. The result of Shapiro–Wilk test turned out that the yield and LAI × SPAD data followed normal distribution – Table 2. And a simple linear regression analysis of LAI × SPAD and yield was presented in Figure 5A. At booting stage, the result provided a satisfactory linear fitting equation between yield and LAI × SPAD with a relatively high R^2^ value (*R*^2^ = 0.627^∗∗^). However, at heading stage, an obviously lower R^2^ value (*R*^2^ = 0379^∗∗^) was found compared with booting stage.

**Table 2:** The statistical description and Shapiro–Wilk test results of LAI × SPAD, abundance and yield.

		Observation plots	Minimum value	Maximum value	Mean value	*p-*Value	Coefficient of variation
LAI × SPAD	Booting stage	22	87.66	201.74	148.79	0.079	23.25%
	Heading stage	22	86.74	233.92	164.96	0.076	27.88%
Leaf abundance	Heading stage	23	0.64	1.00	0.93	0.000	–
Panicle abundance	Heading stage	23	0.00	0.33	0.06	0.000	–
Yield		23	2.70	4.46	3.61	0.948	11.95%

*p-Value is the result of Shapiro–Wilk test.*

### Correlations of Vegetation Index With Yield and LAI × SPAD

**FIGURE 6:**
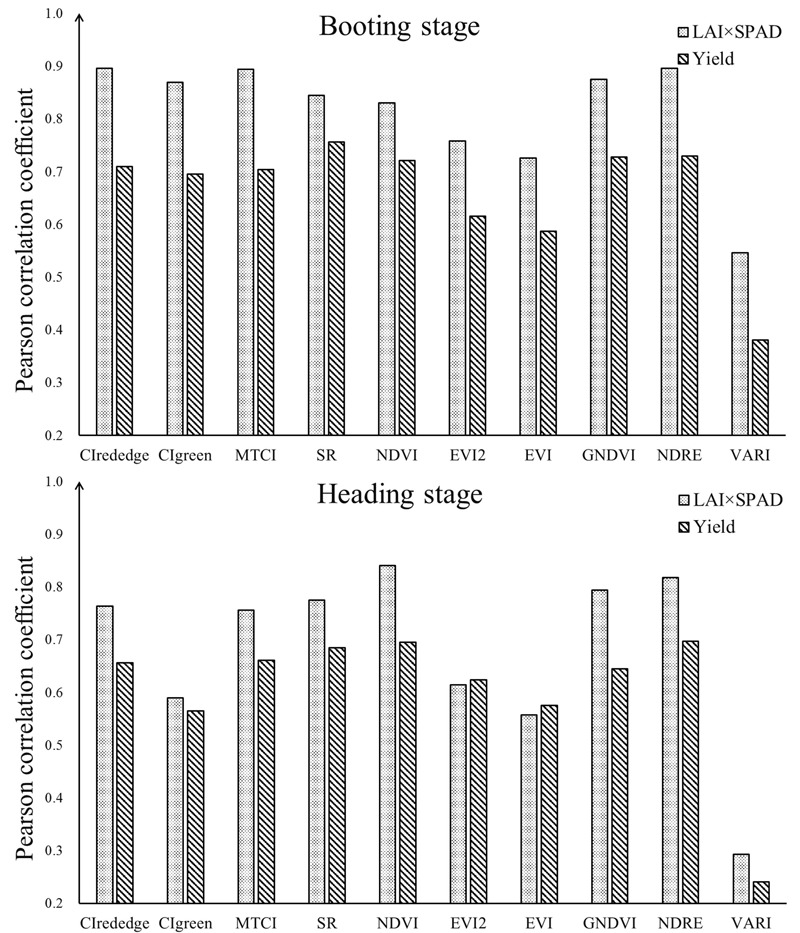
The contrast between Pearson correlation coefficients of VI vs. yield and VI vs. LAI × SPAD at booting stage and heading stage.

Vegetation index calculated from reflectance of different wavebands could be used to estimate rice growth parameters and thus has a good potential to predict the rice yield. To compare the relationships of VI to LAI × SPAD and yield, we performed a correlation analysis at booting and heading stages. The result of correlation analysis indicated that the VIs were correlated positively to LAI × SPAD and yield except VARI, with 0.01 significance levels at two individual stages – Table 3. On the whole, the Pearson correlation coefficients (r) of VI and LAI × SPAD were obviously higher than that of VI and yield at these two stages. Compared with heading stage, most VIs exhibited better correlation with yield and LAI × SPAD at booting stage. Generally, the VIs which had better correlation with LAI × SPAD also produced higher r values with yield at both two stages – Figure 6. At booting stage, SR and NDRE showed most strong correlations with yield (r was 0.756 and 0.730 respectively), and the r values of most VIs were above 0.7 except CI_green_, EVI2 and EVI. Where it came to heading stage, the situation was different, the r values of all VIs were below 0.7. Among the test indices, NDRE and NDVI had the highest r values with yield (r was 0.697 and 0.695 respectively). In addition, the relationships of VARI vs. yield appeared obvious extremely weak correlation at both booting and heading stage. For the most relevant VI with yield at each stage (SR at booting stage and NDRE at heading stage), their correlation with LAI × SPAD had little difference (r was 0.844 and 0.818 respectively), but the result was obviously different when correlated with yield (r was 0.756 and 0.697 respectively).

**Table 3:** The Pearson correlation coefficients of VI with yield and LAI × SPAD at booting and heading stage.

	Growth stage	SR	NDRE	GNDVI	NDVI	CI_rededge_	MTCI	CI_green_	EVI2	EVI	VARI
Yield	Booting stage	0.756^∗∗^	0.730^∗∗^	0.728^∗∗^	0.721^∗∗^	0.710^∗∗^	0.704^∗∗^	0.696^∗∗^	0.616^∗∗^	0.586^∗∗^	-0.381
	Heading stage	0.685^∗∗^	0.697^∗∗^	0.644^∗∗^	0.695^∗∗^	0.656^∗∗^	0.661^∗∗^	0.565^∗∗^	0.624^∗∗^	0.575^∗∗^	-0.386
LAI × SPAD	Booting stage	0.844^∗∗^	0.896^∗∗^	0.875^∗∗^	0.830^∗∗^	0.896^∗∗^	0.894^∗∗^	0.869^∗∗^	0.757^∗∗^	0.726^∗∗^	-0.546^∗∗^
	Heading stage	0.774^∗∗^	0.818^∗∗^	0.794^∗∗^	0.841^∗∗^	0.763^∗∗^	0.756^∗∗^	0.589 ^∗∗^	0.614^∗∗^	0.557^∗∗^	-0.293

*^∗∗^Correlation is significant at the 0.01 level (two-tailed).*

### Spectral Mixture Analysis in Rice Field

In consideration of the impact of mixed pixel, SMA was applied to the UAV image obtained at rice heading stage. According to the ground measured spectra, there was obvious spectral difference in various endmembers – Figure 4. In the top layer, both leaf and panicle spectra appeared the peak and valley configuration as that of other typical vegetation spectra characteristics. Nevertheless, leaf reflectance was a little higher than panicle reflectance in blue bands (7 vs. 5%) and it is much higher than panicle reflectance in NIR bands. On the contrary, leaf reflectance was a bit lower than panicle reflectance in red bands. Compared to the top layer, both leaf reflectance and panicle reflectance were low in the bottom layer, but leaf reflectance was still higher than panicle reflectance in NIR bands. As for soil endmember, the reflectance decreased at all wavelengths with soil moisture increasing.

**FIGURE 7:**
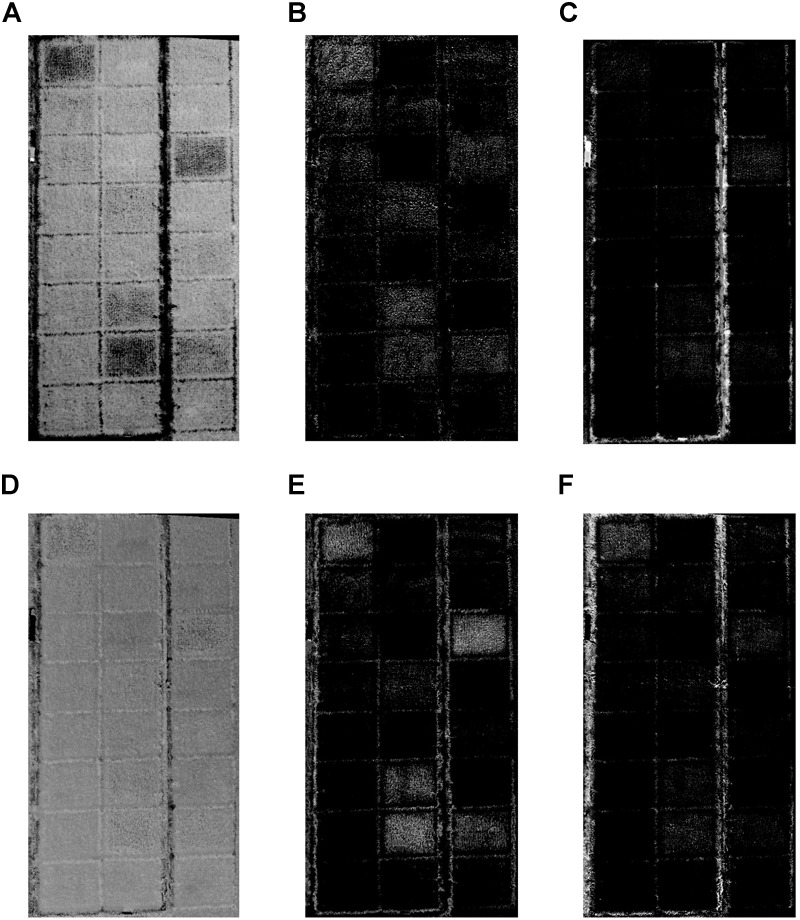
The abundance images of **(A)** top layer leaf, **(B)** top layer panicle, **(C)** dry soil, **(D)** bottom layer leaf, **(E)** bottom layer panicle, and **(F)** wet soil.

In this study, abundance image of each component was obtained using fully constrained least squares linear SMA and the spectra of selected endmember measured by ASD. As shown in Figure 7, significant differences existed in abundance images of different endmembers. On the whole, the brightness of the dry and wet soil abundance images was relatively low. And the bright pixels were mainly clustered in the ridges surrounding the plots in these two abundance images, while the pixels located at rice growing area were really dark. For the other four abundance images, leaf abundance images were obviously brighter than panicle abundance images both in top and bottom layers which was fully corresponded to the actual occurrence in paddy field. Noted that obvious brightness heterogeneity was existed among different plots in the images, and such heterogeneity patterns were quite different in panicle abundance images which indicated the uneven emergence of rice panicles.

In consideration of endmember selection, the sum of panicle abundance in top layer and bottom layer was calculated as panicle abundance (Abd_P_), and the leaf abundance (Abd_L_) was obtained in the same way. A normal distribution test was also used to Abd_L_ and Abd_P_ data, the result of statistical description and Shapiro–Wilk test was presented in Table 2. Evidently, Abd_L_ concentrated near 1, while Abd_P_ concentrated near 0. The aggregation effect of abundance data was more apparent in the fit plot of yield with Abd_L_ and Abd_P_ – Figure 5B.

### Rice Yield Estimation Using Vegetation Index and Abundance Data

At heading stage, the uneven emergence of panicles may influence the accuracy of rice yield estimation. Since panicle abundance and leaf abundance were the indicators of how much panicle had been emerging, in our proposed approach the VIs were incorporated with the information of plot-level panicle abundance (Abd_P_) and leaf abundance (Abd_L_) to estimate the yield of rice. In this part, yield was firstly correlated with VI, VI × Abd_L_, and VI × Abd_P_ respectively, and the Pearson correlation coefficients (r) were compared – Table 4. Generally, after multiplied by Abd_L_ or Abd_P_, most VIs produced relatively higher r values with yield than VI alone. The result of correlation analysis indicated that the VI × Abd_L_ was correlated positively with yield; on the contrary, VI × Abd_P_ showed a negative correlation. And the correlation is numerically higher in VI × Abd_P_ and yield than in VI × Abd_L_ and yield. In particular, VARI showed a really weak correlation with yield at heading stage (r was -0.386), and there was even no correlation in VARI × Abd_L_ and yield. However, after multiplied by Abd_P_, VARI showed a relatively strong correlation (r was -0.734).

**Table 4:** The Pearson correlation coefficients of yield with VI, VI×Abd_L_, VI×Abd_P_, and VI×Abd_L-P_ at heading stage.

	VI	VI×Abd_L_	VI×Abd_P_	VI×Abd_L-P_
NDRE	0.697**	0.751**	-0.775**	0.770**
NDVI	0.695**	0.759**	-0.759**	0.761**
SR	0.685**	0.724**	-0.785**	0.747**
MTCI	0.661**	0.708**	-0.783**	0.736**
CI_rededge_	0.656**	0.698**	-0.786**	0.725**
GNDVI	0.644**	0.753**	-0.765**	0.767**
EVI2	0.624**	0.726**	-0.752**	0.745**
EVI	0.575**	0.710**	-0.747**	0.738**
CI_green_	0.565**	0.609**	-0.796**	0.643**
VARI	-0.386	-0.081	-0.734**	0.183

*^∗∗^Correlation is significant at the 0.01 level (two-tailed).*

For further analysis, regression analysis has been used, and three linear relationship were developed using 23 samples: (1) yield vs. VI, (2) yield vs. VI × Abd_L_ and (3) yield vs. VI × Abd_P_. Adjusted R^2^, RMSE and *p*-value were obtained in SPSS – Table 5. For all tested indices, multiplying abundance data (VI × Abd_P_ and VI × Abd_L_) significantly increased the goodness of fit with yield, and using VI × Abd_P_ to regress with rice yield was more accurate than using VI × Abd_L_ with lower RMSE and higher Adjusted R^2^ values. Among the Abd_L_ multiplied indices (VI × Abd_L_), NDVI × Abd_L_ had the best goodness of fit with yield (Adjusted R^2^ was 0.555), while CI_green_ × Abd_P_ for Abd_P_ multiplied indices (Adjusted R^2^ was 0.615). Whereas the saturation phenomenon of NDVI, the GNDVI × Abd_L_ was also taken into account. As shown in Figure 5C, the linear fitting result of yield and these three indices (NDVI × Abd_L_, GNDVI × Abd_L_ and CI_green_ × Abd_P_) was acceptable with R^2^ above 0.56, and CI_green_ × Abd_P_ produced the highest R^2^ (R^2^ was 0.633). However, the saturation phenomenon of NDVI was still existed in NDVI × Abd_L_, and the CI_green_ × Abd_P_ data concentrated near 0 which caused a phenomenon similar to saturation. There was no obvious saturation in the fit plot of yield and GNDVI × Abd_L_.

**Table 5:** Regression analysis of yield with VI, VI×Abd_L_, VI×Abd_P_, and VI×Abd_L-P_ at heading stage.

	Adjusted R^2^				RMSE				*p*-Value			
	
	VI	VI×Abd_L_	VI×Abd_P_	VI×Abd_L-P_	VI	VI×Abd_L_	VI×Abd_P_	VI×Abd_L-P_	VI	VI×Abd_L_	VI×Abd_P_	VI×Abd_L-P_
NDRE	0.461	0.543	0.582	0.574	0.317	0.291	0.279	0.282	0.000	0.000	0.000	0.000
NDVI	0.456	0.555	0.556	0.569	0.317	0.288	0.287	0.283	0.000	0.000	0.000	0.000
SR	0.443	0.501	0.599	0.559	0.322	0.304	0.273	0.286	0.000	0.000	0.000	0.000
MTCI	0.410	0.477	0.595	0.537	0.331	0.312	0.275	0.293	0.001	0.000	0.000	0.000
CI_rededge_	0.403	0.463	0.600	0.534	0.333	0.316	0.273	0.294	0.001	0.000	0.000	0.000
GNDVI	0.387	0.546	0.566	0.523	0.338	0.291	0.284	0.298	0.001	0.000	0.000	0.000
EVI2	0.360	0.504	0.545	0.519	0.345	0.304	0.291	0.299	0.001	0.000	0.000	0.000
EVI	0.299	0.481	0.538	0.502	0.361	0.311	0.293	0.304	0.004	0.000	0.000	0.000
CI_green_	0.286	0.341	0.615	0.385	0.364	0.350	0.267	0.338	0.005	0.002	0.000	0.001
VARI	0.108	-0.041	0.518	-0.012	0.407	0.440	0.299	0.434	0.069	0.714	0.000	0.403

The Abd_P_ multiplied indices (VI × Abd_P_) could produce higher R^2^ when regressed with yield, but was subjected to saturation (the value was closed to 0). As for Abd_L_ multiplied indices, they were not susceptible to saturation although their R^2^ was relatively lower. And Figure 5B showed that Abd_L_ related positively with yield but Abd_P_ showed a negatively correlation. Therefore, the difference of Abd_L_ and Abd_P_ (Abd_L-P_) was next used to incorporate with VI. In this way, the correlation analysis and regression analysis were also applied to the relationship of VI × Abd_L-P_ and yield – Tables 4, 5. Compared with VI, VI × Abd_L-P_ acquired better goodness of fit when regressed with yield, Adjusted R^2^ could reach 0.574 and RMSE below 0.282 (NDRE × Abd_L-P_). And for the best two indices (NDRE × Abd_L-P_ and GNDVI × Abd_L-P_), there was no obvious saturation existing in fit plot – Figure 5D. Then on the base of these, leave one out cross-validation approach was utilized to obtain the final yield estimation model. The specific estimation formulas and the goodness of fit between measured yield and estimated yield was shown in Figure 5E.

## Discussion

The primary purpose of this study was to improve the accuracy of rice yield estimation at heading stage based on the UAV data. Vegetation index (VI) and SMA were incorporated to construct new yield estimation algorithm.

In this paper, the same two sets of data were acquired at booting and heading stage respectively, including ground measured data (LAI × SPAD) and UAV data. At these two stages, rice gradually completed the transformation from vegetative growth to reproductive growth. The interval between each two stages was around 2 weeks, and rice had similar growth status except the influence of panicle. At booting stage, the rice plant flourished but with no panicle appearing in canopy. On the contrary, the panicle gradually emerged in canopy at heading stage with the growth of rice plant. At these two stages, the changes of rice leaves were not obvious – Figure 3.

First of all, the ground measured LAI × SPAD was correlated with rice yield directly at the two individual stages. Before regression analysis, LAI × SPAD data and yield data have passed the normal distribution test. As shown in Figure 5A, the goodness of fit between LAI × SPAD and yield at booting stage was better than that at heading stage. And this result revealed that the booting stage had better predictive ability for rice yield based on ground measured data. The reason for this was probably that the panicle began to emerge at heading stage. Sun et al. (2010) found that the appearance of panicle may lead to the changes of canopy spectral reflectance and thus the predictive ability for yield decreased during the later stages of rice growth. In our study, the LAI × SPAD was calculated from ground measured LAI and SPAD, and the LAI data was obtained by a Sunscan canopy analysis system. According to the principle of the used SunScan instrument, each organ of rice plant (including leaf, panicle, and stem) contributed to the LAI value. Compared with booting stage, the LAI value of heading stage contained extra panicle information. Actually, the LAI × SPAD which derived from green leaves was associated with rice yield (Liu et al., 2017). Therefore, the booting stage had better predictive ability for rice yield than heading stage based on ground measured LAI × SPAD.

The plot-level VI derived from UAV data was also correlated with rice yield. We chose 10 widely used VIs which were successfully applied in estimating vegetation grow-related parameters such as chlorophyll content, LAI, vegetation fraction and grain yield. The similar result was found that the Pearson correlation coefficient (r) between VI and yield at booting stage was mostly higher than that at heading stage – Table 3. And the correlation analysis was also applied to analyze the relationship between VI and ground measured LAI × SPAD. Although the correlation between VI and LAI × SPAD had a bit difference at these two stages, most VIs were well-correlated with LAI × SPAD (r above 0.75). Moreover, the correlation of VI and yield was consistent with the correlation of VI and LAI × SPAD – Figure 6. This result revealed that the VI which had a better good correlation with LAI × SPAD probably produced a higher r value with yield. The LAI × SPAD was, so to speak, a bridge between VI and yield. VI derived from UAV data had correlation with LAI × SPAD and thus had a potential to estimate yield. As shown in Table 3, the satisfactory correlation could be found between VI and LAI × SPAD at both two stages, but the LAI × SPAD correlated VIs showed obviously different correlation with yield at booting and heading stage. As mentioned above, the ground measured LAI × SPAD contained the extra panicle information at heading stage. In this way, the VI, correlated with LAI × SPAD, may also be affected by panicle emergence which lead to the decrease of yield estimation based on UAV data at heading stage.

At heading stage, the plot-level VI was calculated from mixed components including different proportion of panicles, using VI alone for yield regression may introduce unexpected uncertainties. Therefore, the SMA was utilized to improve the predictive ability of VI in rice yield estimation at heading stage.

At this stage, the rice plant had really luxuriant growth and the paddy field was almost covered by rice plants. The canopy of rice was comprised of leaf and panicle – Figure 3. And the background of field was mainly soil (wet soil and dry soil), because the water was drained off. In addition, the field management of rice was strict and there were no other plants affecting the growth of rice such as weed. Therefore, six endmembers were selected, including top layer leaf (TL), bottom layer leaf (BL), top layer panicle (TP), bottom layer panicle (BP), dry soil (DS), and wet soil (WS). Based on fully constrained least squares linear SMA, the abundance images of six mainly endmembers in paddy field were derived. Of the six abundance images we obtained, the brightness of dry and wet soil abundance images was relatively low. And compared to panicle abundance image, the leaf abundance image was obviously brighter both in top layer and bottom layer. This was consistent with the actual situation of paddy field. At heading stage, rice leaves grew lushly and the leaves occupied the largest proportion in paddy field. Due to the different nitrogen treatments applied in 24 plots, the growth situation of rice plant in different plots significantly varied from each other even at the same growth stage. It is observed that there was obvious difference among different plots in leaf abundance images and panicle abundance images – Figure 7. In consideration of endmember selection, the sum of panicle abundance in top layer and bottom layer was calculated as panicle abundance (Abd_P_), and the sum of leaf abundance in top layer and bottom layer was calculated as leaf abundance (Abd_L_). The result of analysis indicated that neither Abd_L_ nor Abd_P_ followed normal distribution and Abd_L_ concentrated near 1, while Abd_P_ concentrated near 0. This numerical distribution characteristic was in accordance with the sum-to-one constraint condition in fully constrained least squares linear SMA. Accordingly, the abundance data was not adequate for yield estimation due to its aggregation effect – Figure 5B.

Note that, the LAI × SPAD of green leaf was associated with rice yield closely and the VI obtained from UAV data had a good correlation with LAI × SPAD at booting and heading stage. However, the LAI × SPAD derived from VI contained extra panicle information at heading stage which decreased the predictive ability of VI in rice yield. The abundance data, indeed, implies the proportion information of different components in rice field. In this case, Abd_L_ was incorporated with VI to extract the portion of green leaf in total LAI × SPAD (contained panicle portion) and thus the VI × Abd_L_ was used to estimate rice yield at heading stage. For comparison purpose, the relationship of VI × Abd_P_ and yield was also analyzed. The result of correlation analysis revealed that after multiplied by Abd_L_ or Abd_P_, most VIs produced relatively higher r values with yield than VI alone – Table 3. And the VI × Abd_L_ was correlated positively with yield, while VI × Abd_P_ showed a negative correlation. According to the sum-to-one constraint condition of fully constrained SMA, the correlation of VI × Abd_L_ and yield was opposite to that of VI × Abd_P_ and yield. Obviously, the VI × Abd_L_, reflected the LAI × SPAD contributed by green leaf, had a positive correlation with yield. The result of analysis also revealed that VI × Abd_P_ exhibited a better correlation with yield than VI × Abd_L_ did. However, as shown in Figure 5C, VI × Abd_P_ concentrated on 0 and the aggregation effect of VI × Abd_P_ produced a relatively higher R^2^. The reason was that Abd_P_ was close to 0 and the value of VI × Abd_P_ deeply depended on Abd_P_. Although there was no aggregation phenomenon in VI × Abd_L_, the saturation of VI itself still existed in its corresponding VI × Abd_P_ (NDVI and NDVI × Abd_L_).

The Abd_L_ multiplied indices (VI × Abd_L_) and Abd_P_ multiplied indices (VI × Abd_P_) both had its advantages and disadvantages, VI × Abd_P_ could produce higher r but subjected to aggregation, while VI × Abd_L_ was not affected by aggregation but produced relatively lower r. Therefore, Abd_L_ and Abd_P_ were combined together to give full play to the advantages of the two. In consideration of that Abd_L_ correlated positively with yield and Abd_P_ correlated negatively with yield – Figure 5B, the difference of Abd_L_ and Abd_P_ was calculated as Abd_L-P_ and the relationship between VI × Abd_L-P_ and yield was analyzed. The regression analysis showed that the goodness of fit in VI × Abd_L-P_ and yield was better than that in VI and yield (with higher Adjusted R^2^) – Table 5. However, not all VIs had better regression results with yield after multiplied by Abd_L-P_ (such as CI_green_ × Abd_L-P_ and VARI × Abd_L-P_). And the VI × Abd_L-P_ was influenced by the VI itself, if the VI had a poor correlation with yield, the corresponding VI × Abd_L-P_ may also show a bad correlation with yield. And the result of regression analysis in VI × Abd_L-P_ and yield was better than that in VI × Abd_L_ and yield but worse than that in VI × Abd_P_ and yield. It was important to note that there was no obvious aggregation effect in VI × Abd_L-P_ – Figure 5D. The Abd_L-P_ multiplied indices (VI × Abd_L-P_) combined the advantages of VI × Abd_L_ and VI × Abd_P_ and performed a better goodness of fit with no obvious aggregation effect in the relationship of VI × Abd_L-P_ and yield. Among all the tested VI × Abd_L-P_, two indices which had highest Adjusted R^2^ were selected (NDRE × Abd_L-P_ and GNDVI × Abd_L-P_) and then leave one out cross-validation approach was utilized to obtain the final yield estimation model – Figure 5E. The result proved that NDRE and GNDVI multiplied by Abd_L-P_ could accurately estimate the rice yield at heading stage with R^2^ reaching 0.6 and estimation errors below 10%.

In this study, we developed a new approach to estimate rice yield at heading stage using the integration of VI and abundance information retrieved from the UAV image. The approach was simple but it given some significant enlightenment for the yield estimation of grain crop like rice. In the application of RS, the impact of spectral mixture must be considered and this is also important to UAV RS technology which has a really high spatial resolution. And the SMA is a good way to get rid of the influence of different spectral components contained in remotely sensed images. Although the endmembers proposed in our approach were limited to rice yield estimation at heading stage, this work may offer a theoretical framework for yield estimation in grain crops which have obvious grain with significantly different spectra from their leaves. In the future study, we will try to apply this approach to satellite data and in other crops.

## Conclusion

In this study, we developed an approach to improve the estimation of rice yield at heading stage using UAV-based Vegetation Index and abundance data. Compared with booting stage, a relatively weaker relationship between VI and rice yield was found at heading stage. The reason was the uneven emergence of rice panicle at heading stage which caused the decrease of predictive ability of VI for rice yield. In order to improve the accuracy of yield estimation at heading stage, a fully constrained least squares linear spectral mixture method was used to eliminate the influence of the panicle appearance on yield estimation. The abundance images of six mainly endmembers in paddy field was produced based on the six-band UAV image and ground measured spectra, including top layer leaf, bottom layer leaf, top layer panicle, bottom layer panicle, dry soil, and wet soil. The integration of plot-level VI and abundance information can estimate rice yield more accurately than using VI alone. Among the test VIs, NDRE, and GNDVI multiplied by the difference of leaf and panicle abundance were the most accurate for yield estimation in rice under different nitrogen fertilizer treatment with estimation errors below 10%.

## Author Contributions

SF conceived of the research ideas and built the infrastructure for the study site to make this research possible. SW provided rice yield and advised on data collection. YG and YP designed the experiments in detail and provided valuable guidance on data analysis. RZ and XW provided important insights and suggestions on this research from the perspective of agronomists. BD performed the majority of the data processing and provided the writing of this paper. All authors read and approved the final manuscript and significant contributions to this manuscript.

## Conflict of Interest Statement

The authors declare that the research was conducted in the absence of any commercial or financial relationships that could be construed as a potential conflict of interest.

## Footnotes

1 https://en.wikipedia.org/wiki/Remote_sensing
